# Supplementary material for: Residual dynamics learning for trajectory tracking for multi-rotor aerial vehicles
Source: Sci Rep. 2024 Jan 22;14:1858. doi: 10.1038/s41598-024-51822-0 (PMC10810356; doi:10.1038/s41598-024-51822-0)
Supplement: Supplementary file 2 — Supplementary Information. [file 41598_2024_51822_MOESM2_ESM.docx]

**Title**: Residual Dynamics Learning for Trajectory Tracking for Multi-rotor Aerial Vehicles

**Short legend:** This video provides a general overview of the proposed technique of residual dynamics learning by considering reference trajectory tracking in a cluttered environment as an example scenario.
